# Supplementary material for: Identifying clinical features in primary care electronic health record studies: methods for codelist development
Source: BMJ Open. 2017 Nov 22;7(11):e019637. doi: 10.1136/bmjopen-2017-019637 (PMC5719324; doi:10.1136/bmjopen-2017-019637)
Supplement: Supplementary file 1 [file bmjopen-2017-019637supp001.pdf]

Online only supplementary material

Table A 1 List of potential codes for inclusion in a list to identify patients with shortness of breath. 'sob' is the binary variable denoting shortness of breath (1: include; 0: exclude)

medcode	desc	sob	Reason for exclusion
**735**	[D]Breathlessness	1	
**741**	[D]Shortness of breath	1	
**820**	[D]Tachypnoea	0	Abnormally rapid breathing, not breathlessness
**982**	[D]Apnoea	0	Absence of breathing, not breathlessness
**1429**	Breathlessness	1	
**2506**	[D]Sleep apnoea syndrome	0	Absence of breathing, not breathlessness
**2563**	[D]Respiratory distress	1	
**2575**	Short of breath on exertion	1	
**2737**	Respiratory distress syndrome	1	
**2931**	Difficulty breathing	1	
**3007**	Newborn transitory tachypnoea	0	Abnormally rapid breathing, not breathlessness
**3092**	[D]Dyspnoea	1	
**4822**	Shortness of breath	1	
**5175**	Breathlessness symptom	1	
**5349**	Shortness of breath symptom	1	
**5896**	Dyspnoea - symptom	1	
**6326**	Breathless - moderate exertion	1	

**6434**	Paroxysmal nocturnal dyspnoea	1	
**7000**	O/E - dyspnoea	1	
**7534**	O/E - respiratory distress	1	
**7603**	Sleep apnoea	0	Absence of breathing
**7683**	Breathless - lying flat	1	
**7932**	Breathless - mild exertion	1	
**8148**	Obstructive sleep apnoea	0	Absence of breathing
**9089**	Orthopnoea symptom	1	
**9297**	[D]Respiratory insufficiency	1	
**10114**	O/E - tachypnoea	0	Abnormally rapid breathing, not breathlessness
**11451**	[D]Orthopnoea	1	
**12474**	SOBOE	1	
**18116**	Nocturnal dyspnoea	1	
**19346**	No breathlessness	0	Negation of breathlessness
**19426**	MRC Breathlessness Scale: grade 3	1	
**19427**	MRC Breathlessness Scale: grade 2	1	
**19429**	MRC Breathlessness Scale: grade 5	1	
**19430**	MRC Breathlessness Scale: grade 4	1	
**19432**	MRC Breathlessness Scale: grade 1	1	
**19576**	Apnoea of newborn	0	Absence of breathing
**20438**	[D]Syndrome sleep apnoea	0	Absence of breathing
**20748**	Obstructive sleep apnoea	0	Absence of breathing
**21801**	Breathlessness NOS	1	

**22094**	Short of breath dressing/undressing	1	
**23779**	Sleep apnoea	0	Absence of breathing
**23924**	Scoline apnoea	0	Absence of breathing
**24848**	Adult respiratory distress syndrome	1	
**24889**	Breathless - strenuous exertion	1	
**26684**	Perinatal respiratory distress NOS	0	Related to pregnancy not pathology
**26871**	Primary sleep apnoea of newborn	0	Absence of breathing
**31143**	Breathless - at rest	1	
**31913**	O/E - hyperpnoea	0	Increased depth and rate of breathing, not breathlessness
**36301**	[D]Hypersomnia with sleep apnoea	0	Absence of breathing
**37667**	Apnoea alarm monitoring	0	Absence of breathing
**37704**	O/E - orthopnoea	1	
**42287**	Borg Breathlessness Score: 6 severe (+)	1	
**48539**	[D]Insomnia with sleep apnoea	0	Absence of breathing
**53771**	Dyspnoea on exertion	1	
**54594**	[M]Me**sob**lastic nephroma	0	Contains 'sob' string, but is inappropriate
**57193**	Borg Breathlessness Score: 3 moderate	1	
**57283**	Introduction of tran**sob**turator tape	0	Contains 'sob' string, but is inappropriate

**57678**	Adult respiratory distress syndrome	1	
**57759**	Borg Breathlessness Score: 2 slight	1	
**57903**	CLASP shortness of breath score	1	
**58538**	Fu**sob**acterial necrotising tonsillitis	0	Contains ‘sob’ string, but is inappropriate
**59860**	Borg Breathlessness Score: 4 somewhat severe	1	
**60096**	CLASP shortness of breath score	1	
**64049**	Borg Breathlessness Score: 5 severe	1	
**65353**	Borg Breathlessness Score: 0 none at all	0	Negates breathlessness
**67566**	Borg Breathlessness Score: 9 very, very sev (almost maximal)	1	
**68707**	Borg Breathlessness Score: 1 very slight	1	
**70061**	Borg Breathlessness Score: 7 very severe	1	
**70818**	Borg Breathlessness Score: 0.5 very, very slight	1	
**72334**	Borg Breathlessness Score: 8 very severe (+)	1	
**72704**	[X]Other respiratory distress of newborn	0	Newborn

**73978**	[X]Other apnoea of newborn	0	Absence of breathing, not breathlessness
**93869**	Removal of tran**sob**turator tape	0	Contains ‘sob’ string, but is inappropriate
**97037**	Introduction of tran**sob**turator sling	0	Contains ‘sob’ string, but is inappropriate
**98965**	Urine beta amino i**sob**utyrate level	0	Contains ‘sob’ string, but is inappropriate
**100177**	Berlin questionnaire for sleep apnoea	0	Related to absence of breathing
**101843**	Borg Breathlessness Score: 10 maximal	1	
**Total**		78	

Table A 2 Delphi review of codes for shortness of breath. 1: definitely include; 2: uncertain; 3: definitely exclude

medcode	desc	Reviewer 1 Decision	Reviewer 1 comment	Reviewer 2 decision	Reviewer 2 comment
**735**	[D]Breathlessness	1		1	
**741**	[D]Shortness of breath	1		1	
**1429**	Breathlessness	1		1	
**2563**	[D]Respiratory distress	1		2	I would usually only use this term in children
**2575**	Short of breath on exertion	1		1	
**2737**	Respiratory distress syndrome	1		2	I would usually only use this term in children
**2931**	Difficulty breathing	1		1	
**3092**	[D]Dyspnoea	1		1	
**4822**	Shortness of breath	1		1	
**5175**	Breathlessness symptom	1		1	
**5349**	Shortness of breath symptom	1		1	
**5896**	Dyspnoea - symptom	1		1	
**6326**	Breathless - moderate exertion	1		1	
**6434**	Paroxysmal nocturnal dyspnoea	1		1	
**7000**	O/E - dyspnoea	1		1	
**7534**	O/E - respiratory distress	1		2	
**7683**	Breathless - lying flat	1		1	
**7932**	Breathless - mild exertion	1		1	
**9089**	Orthopnoea symptom	1		1	
**9297**	[D]Respiratory insufficiency	1		2	

**11451**	[D]Orthopnoea	1		1	
**12474**	SOBOE	1		1	
**18116**	Nocturnal dyspnoea	1		1	
**19426**	MRC Breathlessness Scale: grade 3	1		1	
**19427**	MRC Breathlessness Scale: grade 2	1		1	
**19429**	MRC Breathlessness Scale: grade 5	1		1	
**19430**	MRC Breathlessness Scale: grade 4	1		1	
**19432**	MRC Breathlessness Scale: grade 1	1		1	
**21801**	Breathlessness NOS	1		1	
**22094**	Short of breath dressing/undressing	1		1	
**24848**	Adult respiratory distress syndrome	1		2	
**24889**	Breathless - strenuous exertion	1		1	
**31143**	Breathless - at rest	1		1	
**37704**	O/E - orthopnoea	1		1	
**42287**	Borg Breathlessness Score: 6 severe (+)	1		1	I would never use this scale and not sure who would but if it is coded then it would be relevant
**53771**	Dyspnoea on exertion	1		1	
**57193**	Borg Breathlessness Score: 3 moderate	1		1	
**57678**	Adult respiratory distress syndrome	1		1	
**57759**	Borg Breathlessness Score: 2 slight	1		1	

**57903**	CLASP shortness of breath score	1		1	as above
**59860**	Borg Breathlessness Score: 4 somewhat severe	1		1	
**60096**	CLASP shortness of breath score	1		1	
**64049**	Borg Breathlessness Score: 5 severe	1		1	
**67566**	Borg Breathlessness Score: 9 very, very sev (almost maximal)	1		1	
**68707**	Borg Breathlessness Score: 1 very slight	1		1	
**70061**	Borg Breathlessness Score: 7 very severe	1		1	
**70818**	Borg Breathlessness Score: 0.5 very, very slight	1		1	
**72334**	Borg Breathlessness Score: 8 very severe (+)	1		1	
**101843**	Borg Breathlessness Score: 10 maximal	1		1	

Table A 3 Sensitivity analysis: Medical codes used to record shortness of breath in a group of patients in the year before they were diagnosed with lung cancer

medcode	Description	Frequency	Percent	Cum.
**4822**	Shortness of breath	3,226	24.66	24.66
**741**	[D]Shortness of breath	1,455	11.12	35.78
**1429**	Breathlessness	1,116	8.53	44.32
**19427**	MRC Breathlessness Scale: grade 2	1,106	8.46	52.77
**19426**	MRC Breathlessness Scale: grade 3	1,010	7.72	60.49
**5349**	Shortness of breath symptom	816	6.24	66.73
**5175**	Breathlessness symptom	785	6.00	72.73
**19430**	MRC Breathlessness Scale: grade 4	764	5.84	78.57
**5896**	Dyspnoea - symptom	437	3.34	81.91
**2575**	Short of breath on exertion	415	3.17	85.09
**3092**	[D]Dyspnoea	395	3.02	88.10
**19432**	MRC Breathlessness Scale: grade 1	332	2.54	90.64
**6326**	Breathless - moderate exertion	261	2.00	92.64
**19429**	MRC Breathlessness Scale: grade 5	189	1.44	94.08
**2931**	Difficulty breathing	187	1.43	95.51
**12474**	SOBOE	166	1.27	96.78
**7932**	Breathless - mild exertion	142	1.09	97.87
**735**	[D]Breathlessness	66	0.50	98.37
**7000**	O/E - dyspnoea	49	0.37	98.75
**57903**	CLASP shortness of breath score	44	0.34	99.08
**31143**	Breathless - at rest	39	0.30	99.38

**7683**	Breathless - lying flat	22	0.17	99.55
**6434**	Paroxysmal nocturnal dyspnoea	19	0.15	99.69
**21801**	Breathlessness NOS	10	0.08	99.77
**11451**	[D]Orthopnoea	9	0.07	99.84
**9089**	Orthopnoea symptom	8	0.06	99.90
**24889**	Breathless - strenuous exertion	5	0.04	99.94
**18116**	Nocturnal dyspnoea	3	0.02	99.96
**53771**	Dyspnoea on exertion	2	0.02	99.98
**22094**	Borg Breathlessness Score: 10 maximal	1	0.01	99.98
**59860**	Borg Breathlessness Score: 4 somewhat..	1	0.01	99.99
**101843**	Short of breath dressing/undressing	1	0.01	100.00
**Total**	Total	13,081	100.00	

Example R syntax.

Credit: Ben Feakins (Benjamin.Feakins@phc.ox.ac.uk).

```
#============================#
#                            #
#### R CODE FOR APPENDIX ####
#                            #
#============================#

### Set Directory Objects ###
browser.dir <- "/Volumes/PHC/CPRD_data/Browsers"
save.dir <- "~/Desktop"

### Read Data Into R ###
setwd(browser.dir)
medical <- read.delim("medical.txt", header = FALSE,
na.strings = "", stringsAsFactors = FALSE, skip = 1)
names(medical) <- c("medcode", "readcode",
"clinicalevents", "immunisationevents",
"referralevents", "testevents", "readterm",
"databasebuild") # Define headers.


### Define Search Terms ###
search.terms.general <- c("shortness of
breath|sob|pnoea|pnea|puffed|short of
breath|winded|breathless")
search.terms.breath <- c("breath")
search.terms.breath.resid <-
c("short|difficult|labour|labor|distress|insuff")
search.terms.respir <- c("respir")
search.terms.respir.resid <- c("insuff|distress")

### Filtering ###
general <- medical[grepl(search.terms.general,
medical$readterm, ignore.case = TRUE), ]
breath <- medical[grepl(search.terms.breath,
medical$readterm, ignore.case = TRUE), ]
breath <- breath[grepl(search.terms.breath.resid,
breath$readterm, ignore.case = TRUE), ]
respir <- medical[grepl(search.terms.respir,
medical$readterm, ignore.case = TRUE), ]
```

```
respir <- respir[grepl(search.terms.respir.resid,
respir$readterm, ignore.case = TRUE), ]

### Combine Results ###
medcodes <- rbind(general, breath, respir)

### De-Duplicate ###
medcodes <- unique(medcodes)

### Sort ###
medcodes <- medcodes[order(medcodes$medcode), ]

### Remove Codes Not Related to Breathlessness ###
# Further subsetting using grepl().

### Drop Useless Variables ###
medcodes <- medcodes[c("medcode", "readcode")]

### Save a Copy ###
setwd(save.dir)
write.table(medcodes, "sob_library.txt", quote = FALSE,
sep = "\t", na = "", row.names = FALSE)

### Tidying Up ###
rm(browser.dir, save.dir)
rm(search.terms.general, search.terms.breath,
search.terms.breath.resid, search.terms.respir,
search.terms.respir.resid)
rm(medical, general, breath, respir)
rm(medcodes)
```
